# Peptides as Therapeutic Agents: Challenges and Opportunities in the Green Transition Era

**DOI:** 10.3390/molecules28207165

**Published:** 2023-10-19

**Authors:** Giacomo Rossino, Emanuela Marchese, Giovanni Galli, Francesca Verde, Matteo Finizio, Massimo Serra, Pasquale Linciano, Simona Collina

**Affiliations:** 1 Department of Drug Sciences, University of Pavia, Viale Taramelli 12, 27100 Pavia, Italy; giacomo.rossino@unipv.it (G.R.); e.marchese@unicz.it (E.M.); massimo.serra@unipv.it (M.S.); pasquale.linciano@unipv.it (P.L.); 2 Department of Health Sciences, University “Magna Graecia”, Viale Europa, 88100 Catanzaro, Italy

**Keywords:** peptide therapeutics, antimicrobials, antivirals, anticancers, peptide synthesis, green chemistry, solid-phase peptide synthesis, liquid-phase peptide synthesis, microwave-assisted peptide synthesis, chemoenzymatic peptide synthesis

## Abstract

Peptides are at the cutting edge of contemporary research for new potent, selective, and safe therapeutical agents. Their rise has reshaped the pharmaceutical landscape, providing solutions to challenges that traditional small molecules often cannot address. A wide variety of natural and modified peptides have been obtained and studied, and many others are advancing in clinical trials, covering multiple therapeutic areas. As the demand for peptide-based therapies grows, so does the need for sustainable and environmentally friendly synthesis methods. Traditional peptide synthesis, while effective, often involves environmentally draining processes, generating significant waste and consuming vast resources. The integration of green chemistry offers sustainable alternatives, prioritizing eco-friendly processes, waste reduction, and energy conservation. This review delves into the transformative potential of applying green chemistry principles to peptide synthesis by discussing relevant examples of the application of such approaches to the production of active pharmaceutical ingredients (APIs) with a peptide structure and how these efforts are critical for an effective green transition era in the pharmaceutical field.

## 1. Introduction

Peptides have been used in therapy for a century now, since the moment when a team of Canadian researchers discovered the therapeutic potential of insulin for the treatment of type 1 diabetes. The isolation of insulin from living organisms opened the doors for the use of other peptide drugs in hormone replacement therapies. Decades later, the improvement of peptide solid phase synthesis, the development of recombinant technologies, and the discovery of new peptides from natural sources stimulated the scientific world toward the development of peptides as therapeutic agents [1,2,3]. Nowadays, more than 80 approved peptide drugs are available for therapy, and among them, 30 are non-insulin ones, such as oxytocin and vasopressin.

**Figure 1:**
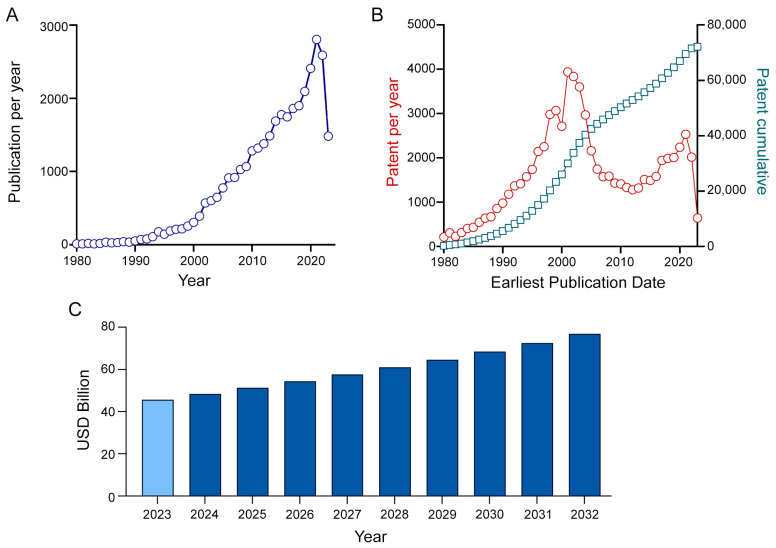
(**A**) The number of scientific publications per year (from 1980 to 2023) about peptides as therapeutic agents. (Source Scopus. Query using ‘peptide’, ‘drugs’, ‘therapeutic agent’ as keywords). (**B**) The number of patents per year (in red) and cumulative (in teal) (from 1980 to 2023) about peptides as therapeutic agents. (Source Espacenet. Query for patent applications belonging to cooperative patent classification CPC: A61K38). (**C**) Peptide therapeutic market size in 2023 and forecasted up to 2032.

Therapeutic applications of peptides represent a research field of growing interest, as testified by the 26 peptides approved as drugs between 2016 and 2022 by the Food and Drug Administration (FDA), with over a total of 315 new drugs approved in the same timeframe [4], with more than 200 peptides in clinical development, and with another 600 peptides undergoing preclinical studies. This trend is further supported by the thriving production in the scientific literature (Figure 1A) and patents (Figure 1B) over the past years, which include excellent reviews on the topic [5,6]. Peptides are currently under development as drugs for treating several pathologies, including microbial infections, obesity, and cancer, and also for developing cell-targeting platforms and improving cell-penetrating properties. Particularly, cell-penetrating peptides are now under investigation as drug delivery tools for anti-cancer, antibacterial, and antiviral therapies [3,6,7].

The relevance of peptides in the pharmaceutical industry is further testified by their impact on the market. In 2022, peptide drugs accounted for 5% of the global pharmaceutical market, with a market size valued at USD 42.05 billion, and they were forecasted to grow at a compound annual growth rate (CAGR) of 10% from 2023 to 2032 (Figure 1C) [8]. The growing use of peptide therapeutics in this period is driven by the expected rising incidence of pathologies such as cancer, viral infections, and metabolic disorders. Moreover, due to the growing number of affected patients in low-income countries, there is an urgent need for economically and environmentally sustainable production methods of such drugs.

Peptide drugs offer several advantages over small molecules. These include heightened target specificity and potency, often reflected in EC50 values within the nanomolar range or even lower [5,6,9,10]. Such specificity typically results in fewer side effects due to reduced interactions with unintended targets. The diversity of side chains in peptides provides a broad spectrum of potential targets. Furthermore, peptides generally exhibit a more predictable metabolism than small molecules. Their metabolites are typically non-toxic, and metabolic interactions, such as cytochrome P450 (CYP) inhibition, are rarer. However, a limitation of the peptide metabolism is that naturally occurring peptides are prone to enzymatic degradation and are swiftly excreted, posing challenges for oral administration [5,6]. To address this, peptide drugs are predominantly administered via the parenteral route. Nonetheless, recent innovations are exploring or utilizing alternative administration methods, including nasal, pulmonary, and transdermal routes [11].

With the aim to further improve the effectiveness and activity of peptides, and to broaden their therapeutic applications, structural modifications have been performed, developing two new important classes of drugs: natural peptide analogues and peptidomimetics [12]. By modifying the side chains and/or the backbone structure, or converting linear peptide chains into cyclic structures, it is possible to maintain and improve not only the high specificity and efficacy of peptide drugs, but also the resistance against proteolysis, ultimately overcoming bioavailability problems [13]. Accordingly, both peptide analogues and peptidomimetics have flourished over the past years and stand out for their own importance as a well-defined category of therapeutics; hence, their discussion is beyond the scope of the present work. For an in-depth discussion on this topic, several excellent publications can be found in the recent literature [14,15,16,17,18,19,20].

Instead, this review focused on therapeutic peptides, providing a comprehensive overview of their potentialities and challenges from a development perspective. In the next paragraphs, after a summary of the most important classes of peptide drugs, we discuss green and eco-friendly approaches to peptide synthesis, considering both well-established procedures and emerging technologies. Indeed, to fully evaluate peptides’ fate as therapeutic tools, synthetic feasibility must be considered, along with pharmacological considerations. Often, traditional synthetic strategies involve the use of toxic, polluting solvents and reagents. Moreover, the amount of produced waste is usually very high [21]. Over the last decades, the attention toward environmental pollution related to industrial processes has grown significantly, and therefore, there have been major efforts to develop greener manufacturing procedures. Particularly, pharmaceutical companies are characterized by very high environmental factors (E factor, i.e., the amount of waste produced per kilogram of product obtained) compared to other manufacturers [22]. Starting from these premises, herein we discuss relevant examples of the application of such approaches to the production of active pharmaceutical ingredients (APIs) with peptide structure on an industrial scale.

## 2. Peptides as Therapeutic Agents

As highlighted in the introduction, peptides have emerged as a significant class of drugs with diverse applications, spanning antimicrobial, anti-tumor, anti-diabetic, and numerous other properties. A comprehensive discussion on this topic was recently presented by Wang et al. [6]. Consequently, in this review, we focus on providing a selection of notable examples, categorised by their therapeutic applications.

### 2.1. Antimicrobial Peptides

**Figure 2:**
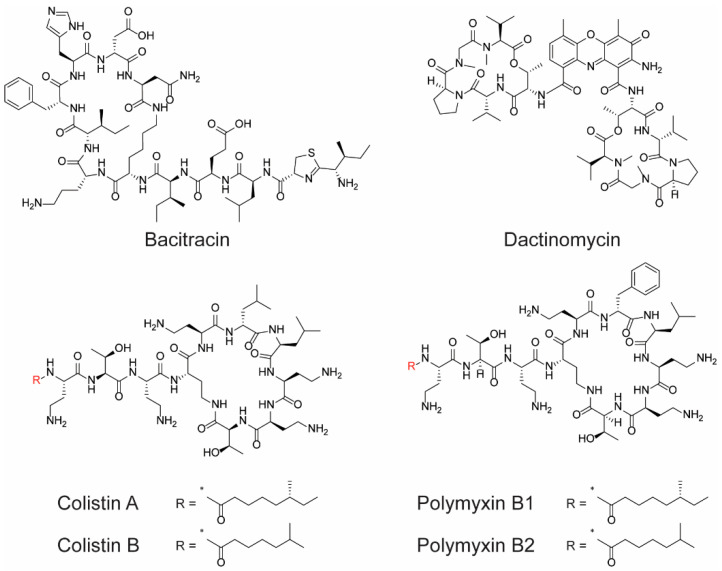
Chemical structure of bacitracin, dactinomycin, colistin A and B, and polymyxin B1 and B2.

The discovery and use of antibiotics containing non-protein polypeptide chains have been a significant advancement in the fight against bacterial infections. These polypeptide antibiotics, which include actinomycins, bacitracin, colistin A, colistin B, polymyxin B1, and polymyxin B2 (Figure 2), have shown effectiveness against both Gram-negative and Gram-positive bacteria.

**Figure 3:**
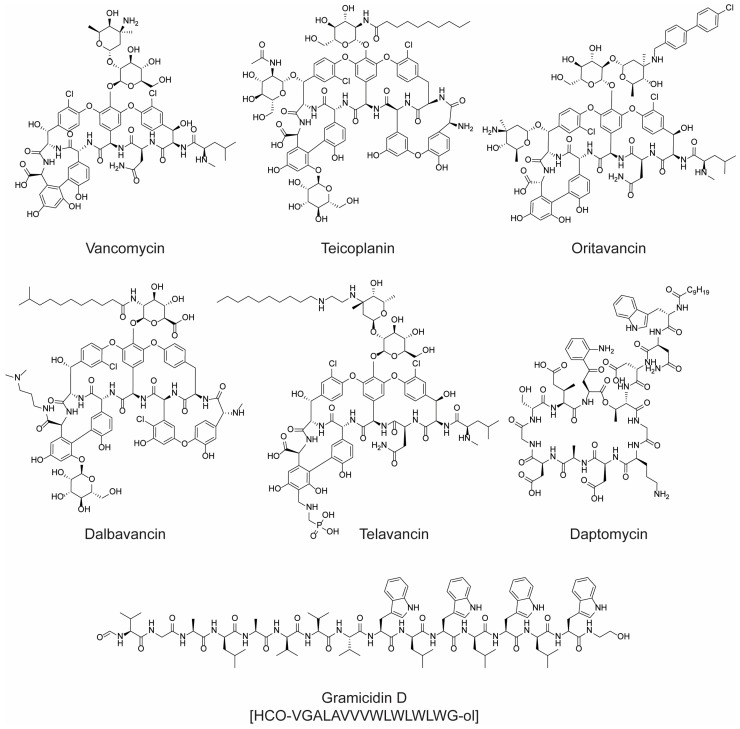
The chemical structure of some marketed AMPs. For gramicidin D and teicoplanin, only the most abundant components of the clinically used mixtures are shown.

Their mechanism of action primarily involves the disruption of the bacterial cell membrane, leading to cell death. Although most polypeptide antibiotics can be considered a milestone in the war against infections, they are not without their limitations. Many of them exhibit cytotoxicity, which restricts their systemic administration, leading to topical application or repurposing for other therapeutic uses, such as cancer treatment (e.g., dactinomycin). Unfortunately, like all antimicrobials, polypeptide antibiotics are also susceptible to the growing challenge of antibiotic resistance (AMR). The rise of AMR poses a significant threat to global health, with projections indicating that, by 2050, infections complicated by AMR could result in the death of 10 million people annually [23,24]. Recognizing the antimicrobial potential of polypeptides and understanding the role of antimicrobial peptides (AMPs) in the innate immune defense of many organisms, researchers have been motivated to explore new peptides with antimicrobial properties [25,26]. Vancomycin, teicoplanin, gramicidin D, daptomycin, oritavancin, dalbavancin, and telavancin (Figure 3), just to name a few, were successfully approved for therapeutic use on humans.

A notable characteristic of these peptides, with the exception of gramicidin D, is their cyclic structure. This cyclic nature is crucial for their efficacy and safety profile. Linear antimicrobial peptides, while they can be potent, often come with significant drawbacks. They tend to exhibit high toxicity when administered systemically, making them unsuitable for many therapeutic applications. Additionally, their linear structure often renders them less stable, making them susceptible to degradation and reducing their effective lifespan in the body. In contrast, cyclic peptides often exhibit enhanced stability and reduced toxicity, making them more favorable candidates for drug development and therapeutic use [27].

**Figure 4:**
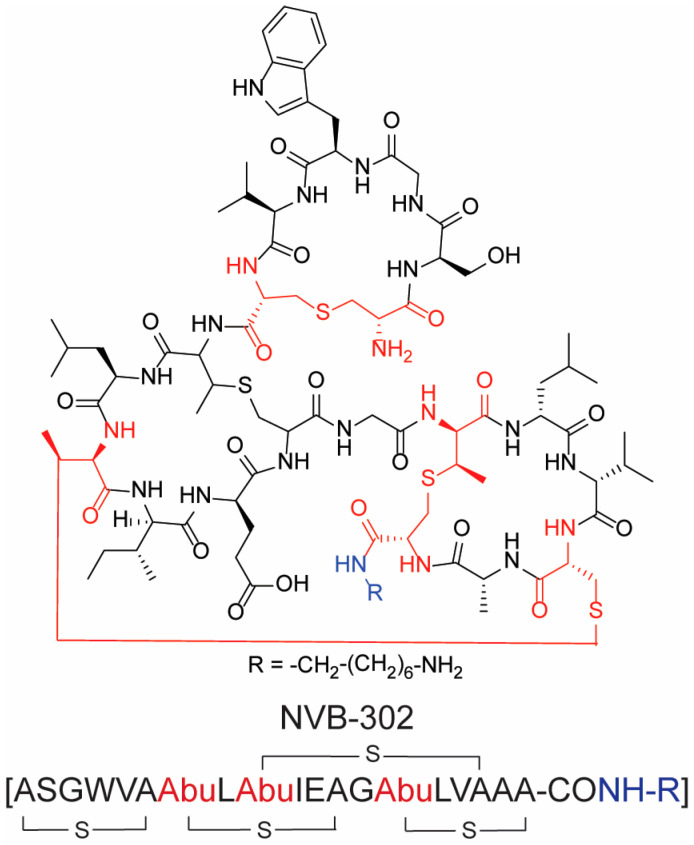
The chemical structure of the lantibiotic NVB-302. The lanthionine and methyllanthionine amino acids involved in the thioether cyclisation are highlighted in red.

Among the antimicrobial cyclic peptides, lantibiotics warrant mention. These represent a novel class of polycyclic natural peptides distinguished by the inclusion of atypical amino acids, notably lanthionine and methyllanthionine, which are cyclized through the formation of a thioether-linker [28]. Lantibiotics are synthesized by Gram-positive bacteria as a defensive strategy against rival Gram-positive bacteria. They predominantly exert their antimicrobial activity by targeting the bacterial cell membrane or inhibiting cell wall synthesis. Given their distinctive structure and mechanism of action, lantibiotics emerge as promising solutions to combat antibiotic-resistant bacterial infections. NVB-302 (Figure 4) is the only lantibiotic that entered a phase I clinical trial for the treatment of *Clostridioides difficile* gut infections [26]. Nevertheless, the clinical trial of NVB-302 was discontinued in 2018, whilst no detail about the outcomes of the study have been disclosed yet [29]. However, several companies have invested in the development of new lantibiotic peptide compounds as broad-spectrum antibacterial agents using novel peptide synthesis platforms.

**Figure 5:**
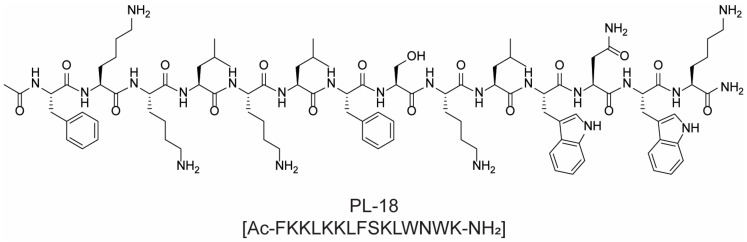
Chemical structure of the antimicrobial polypeptide PL-18.

Several promising antimicrobial polypeptides are currently in the clinical stage. PL-18 (Figure 5) is a polypeptide recently reported for the treatment of bacterial and fungal infections. In vitro and in vivo studies showed quick and strong antimicrobial activity against both Gram-positive and Gram-negative bacterial strains, with MIC values ranging from 1 to 4 μM, whilst its mechanism of action is still undefined [30]. However, in 2022, a single-center, randomized, double-blind, placebo-controlled phase I study was organized in Australia in order to evaluate the safety, tolerability, and pharmacokinetic profiles of the antimicrobial peptide PL-18 through intravaginal suppositories. In particular, the peptide was used in the study to treat colpomycosis, bacterial vaginosis, and mixed vaginitis through a one-to-five dose administration of antimicrobial peptides (escalating dose) [31].

**Figure 6:**
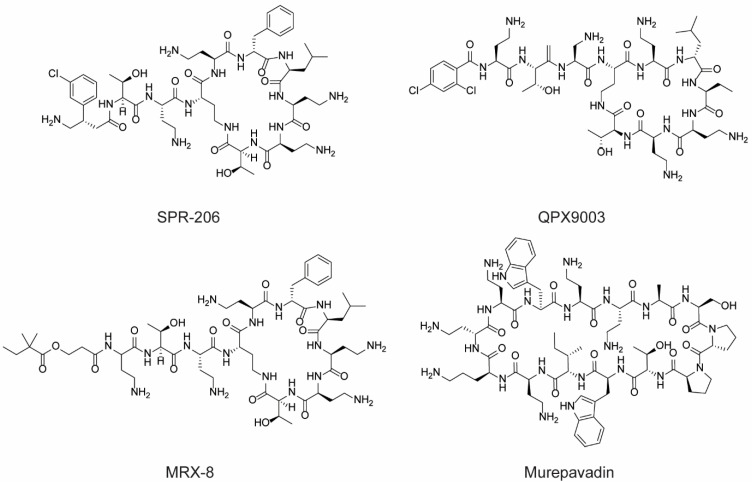
The chemical structure of new polymyxin derivatives SPR 206, QPX 9003, and MRX 8 and of the synthetic cyclic peptide murepavadin, currently in clinical trials.

Among the derivatives of polymyxin, SPR 206, QPX 9003, and MRX 8 warrant mention (Figure 6). SPR 206, also referred to as EVER 206, is a novel polymyxin antibiotic formulated to diminish the renal toxicity frequently observed with polymyxin B and colistin. When administered intravenously or subcutaneously, it has demonstrated efficacy against infections caused by multidrug-resistant (MDR) and extensively drug-resistant (XDR) Gram-negative bacteria, including carbapenem-resistant strains of *Pseudomonas aeruginosa*, *Acinetobacter baumannii*, and Enterobacteriaceae. QPX 9003, also known as BRII 693, targets multidrug-resistant Gram-negative bacterial infections, particularly those caused by *Acinetobacter* and *Pseudomonas*. Its early clinical development is underway in the US. MRX 8 aims to alleviate the nephrotoxicities commonly associated with existing polymyxins. Presently, it is undergoing clinical trials in the US, while preclinical studies are conducted in China to combat multidrug-resistant Gram-negative infections, such as those induced by *Escherichia coli* and *Pseudomonas aeruginosa*. Murepavadin (Figure 6), a 14-amino acid synthetic cyclic peptide, was conceived within the OMPTA (Outer Membrane Protein Targeting Antibiotics) research program. It is a potent antibiotic derived from protegrin I, targeting the beta-barrel protein LptD, a vital outer membrane protein essential for lipopolysaccharide biogenesis. It is currently under clinical evaluation against key Gram-negative ESKAPE pathogens, including *Klebsiella pneumoniae*, *Acinetobacter baumannii*, *Pseudomonas aeruginosa*, and *Enterobacter* spp.

**Figure 7:**
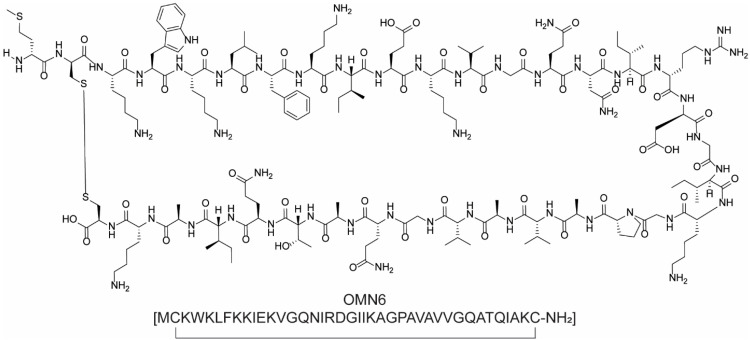
The chemical structure of the antimicrobial peptide OMN6. The amino acidic sequence (using the one letter code) is reported in squared brackets. Disulfide bonds connecting cysteine residues are represented using connection lines in the AA sequence description.

Lastly, OMN6 (Figure 7), a pioneering antimicrobial 40-amino acid cyclic peptide based on cecropin A, produced using advanced genetic-engineering techniques, has gained attention for its potential to combat multidrug-resistant Gram-negative bacteria, such as carbapenem-resistant *Acinetobacter baumannii*. Clinical trials for OMN6 are ongoing in the Netherlands [32].

### 2.2. Antiviral Drugs

Nowadays, viral infections remain one of the main causes of disease deaths. Together with vaccines, antiviral drugs play a relevant role in fighting this kind of illness [33,34,35]. Many peptides from natural sources, including insect and reptile venoms, are able to influence different phases of the viral life cycle. Moreover, short peptides derived from key viral proteins, properly modified (e.g., by inserting lipids and cell-penetrating sequences), have led to the discovery of highly effective antiviral inhibitors. Natural antiviral peptide derivatives often possess excellent resistance to denaturation, degradation, and extreme temperature and pH conditions [36,37,38].

However, it should not be forgotten that viruses are difficult to target, as their evasive infectivity strategies can preclude standard therapeutics.

**Figure 8:**
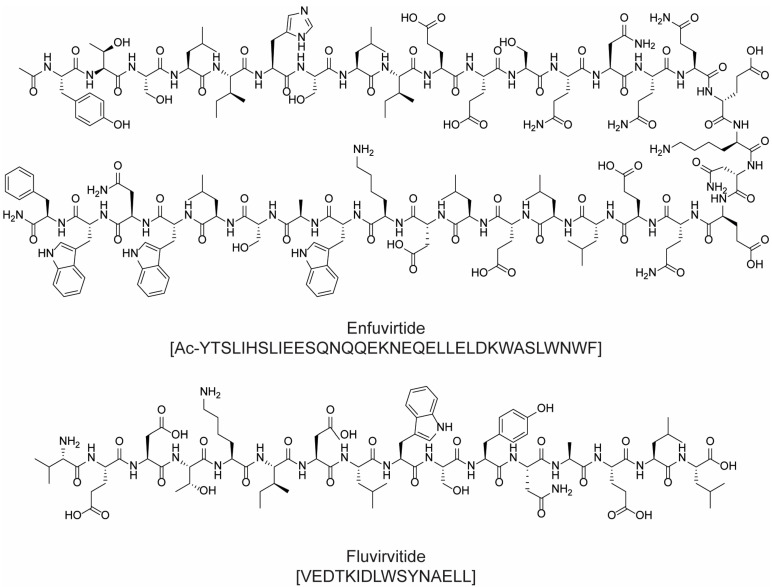
The chemical structure of enfuvirtide and flufirvitide.

As a result of extensive research efforts, antiviral therapies and means of prevention of human immunodeficiency virus (HIV) infection and acquired immune deficiency syndrome (AIDS) have been developed in the past 20 years. Some of the agents developed for the treatment of HIV infection have been shown to inhibit other viruses as well, and the innovative approaches taken in the development of antiretroviral therapy have been applied to develop numerous treatment strategies [39]. Enfuvirtide is the pioneering peptide active against HIV (Figure 8), which was approved in the United States of America in 2003. In detail, it is a biomimetic peptide composed of 36 amino acids, which acts by preventing the fusion between the virus and the target cell, thus avoiding the intracellular uptake of the virus and the subsequent infection.

Influenza infections are mediated through a viral surface glycoprotein called hemagglutinin (HA). After the virus–host membrane fusion, the viral agent is directed to the endosome, where the low pH causes a conformation change in HA and the exposition of its fusion initiation region (FIR); this process leads to fusion with the endosome and the consequential infection. Many inhibitors of the HA refolding at low pH have been reported; however, due to their lack of subspecificity and low resistance barrier, they have never been allowed to reach the preclinical development stage [38]. Flufirvitide (Figure 8) is a peptide sequence of 16 amino acids, obtained from the FIR of HA glycoprotein. This peptide demonstrated the effective inhibition of influenza viral infections, but its mechanism is still only hypothesized. Immunoprecipitation, immunodetection, and fluorescence techniques have been used in order to obtain insights regarding interaction between flufirvitide and HA and the subsequent fusion inhibition [40,41,42].

**Figure 9:**
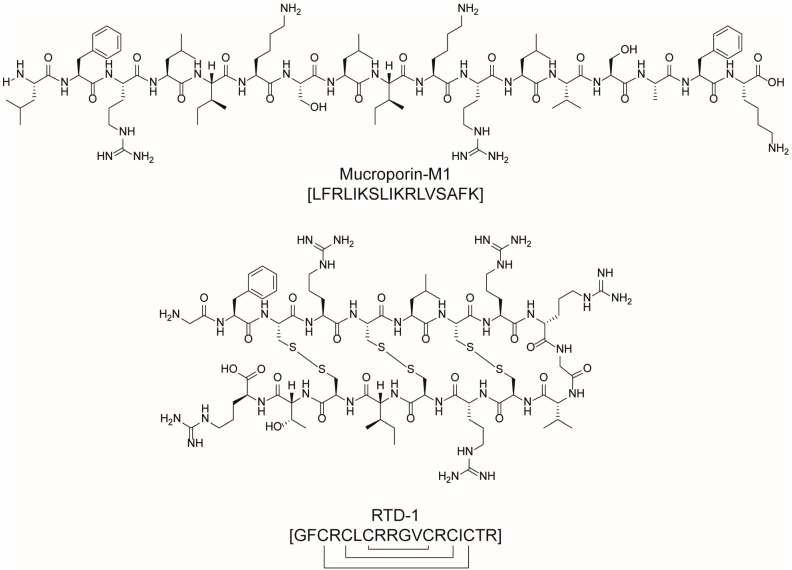
Chemical structures of mucroporin-M1 and RTD-1. The amino acidic sequence (using the one letter code) is reported in squared brackets. Disulphide bonds connecting cysteine residues are represented using connection lines in the AA sequence description.

SARSCoV-2 virus has been the cause of more than 6 million deaths worldwide, and the various therapeutic agents that have been rapidly introduced into clinical trials have largely been based on existing drugs with nonspecific antiviral activity or compounds that are hypothesized to be effective in improving the clinical outcome of patients. Since the beginning of the epidemic, scientists from all over the world have made several efforts to find new drugs to counteract SARSCoV-2. The virus itself has been studied, and potential targets have been identified, with the goal of finding molecules that can interact with them, blocking the spread of the infection. Also in this case, peptides endowed with good antiviral potential have been identified. Among these, peptides able to block the angiotensin-converting enzyme 2 (ACE2) receptor, thus preventing viral recognition and attack, emerged as particularly promising [43,44]. As a title of example, ACE2-blocker mucroporin-M1 (LFRLIKSLIKRLVSAFK, Figure 9) is an engineered peptide analogue carrying four residual mutations with respect to the parent peptide, mucroporin, isolated from the venom of the scorpion *Lychas mucronatus*. As molecular blocker, mucroporin-M1 binds to the ACE2 receptor, allowing for the rupture of the viral envelope [45,46]. Another ACE2-blocker is the natural peptide similar to human lectin-defensin-5 (HD5) (ATCYCRTGRCATRESLSGVCEISGRLYRLCCR), secreted by Paneth cells in Lieberkuhn crypts [46]. The RTD-1 cyclic peptide (GFCRCLCRRGVCRCICTR, Figure 9) from rhesus macaque leukocytes was reported to reduce the pathogenesis of SARS-CoV infection in mice, as evidenced by a substantial reduction in perivascular infiltration and necrotizing bronchiolitis. Together with the increased cytokine levels of interleukin-6, the keratinocyte chemoattractant factor, and the granulocyte colony-stimulating factor in lung cell homogenates, RTD-1 was hypothesized to act as an immunomodulatory effector molecule through the attenuation of the proinflammatory cytokine response in SARS-CoV clearance [46].

**Figure 10:**
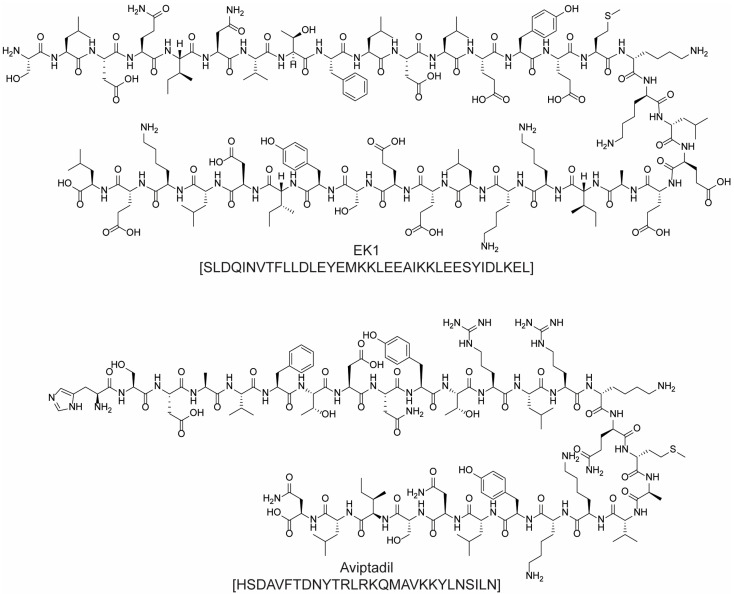
Chemical structures of EK1 and aviptadil. The amino acidic sequence (using the one letter code) is reported in squared brackets.

Antiviral peptides aimed at preventing S-protein-mediated fusion, viral release, and the inhibition of the major protease have been developed as therapeutical strategies for coronavirus infection treatment. Among these, EK1 (Figure 10), a 34-amino acid fusion inhibitory peptide, deserves to be mentioned. This peptide targets the HR1 domains of the spike (S) glycoproteins in several viruses, including SARS-CoV-1, MERS-CoV, and 3 SARS-related SoVs, thus inhibiting virus fusion. A lipid modification was added to EK1 by adding cholesterol or palmitic acid to the peptide through a short spacer. The resulting modification caused stronger membrane interactions and thus higher binding affinity between the peptide and the membrane, increasing the potency of inhibition [46,47]. Lastly, aviptadil (Figure 10) is an injectable synthetic variant of the 28-amino acid vasoactive intestinal polypeptide (VIP) analogue. It was investigated for the treatment of pulmonary sarcoidosis, lung injury, and SARS-CoV-2 acute respiratory disease. The peptide interacts with G-protein-coupled receptors VPAC1, VPAC2, and PACAP-R1 found on various cell types, including alveolar type II (AT-II) cells targeted by the SARS-CoV-2 virus. Aviptadil protects these cells by obstructing cytokines, preventing apoptosis, and enhancing surfactant production, which is crucial for respiratory function. Additionally, it inhibits SARS-CoV-2 replication. While intravenous aviptadil has received emergency use authorization in Georgia, it was denied in the US. Nevertheless, clinical trials for inhalation formulations are ongoing in the USA, Turkey, and Germany.

### 2.3. Anti-Neoplastic Agents

In oncology, peptides have garnered significant attention. They can serve as anti-neoplastic agents, directly inducing the death of cancer cells, or they can be conjugated with chemotherapeutic drugs to selectively target tumor cells [48,49].

**Figure 11:**
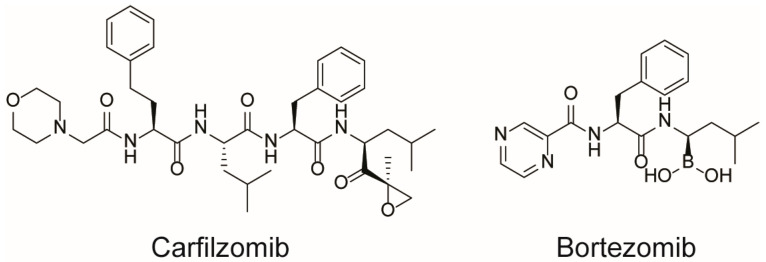
The chemical structure of carfilzomib and of the parent drug bortezomib.

Since the discovery of the first peptides endowed with cytotoxic activity, nature has been a precious resource. Many anticancer peptides (ACPs) are in fact isolated from living organisms or are modifications thereof [50,51]. In this context, carfilzomib (Figure 11) represents a valuable example of success: this second-generation proteasome inhibitor received FDA approval in 2012 for the treatment of patients with relapsed and/or refractory multiple myeloma. Carfilzomib is a tetrapeptide epoxyketone whose structure derives from modifications of epoxomicin, a natural product with anti-inflammatory and proteasome inhibitory activity isolated from the *Actinomycetes* strain. Carfilzomib represents a significant step forward in terms of efficacy and safety compared to the first-generation proteasome inhibitor bortezomib (Figure 11). This is due to its higher selectivity toward proteasomes, which in turn is a consequence of the structural features of epoxomicin and its analogues. The peptide chain selectively and tightly interacts with the binding site of the proteasome, while the epoxyketone covalently bonds to the catalytic threonine residue, thus irreversibly blocking the activity of the β5 subunit [52].

This unique mechanism of action is a prominent example of the results achieved over recent years in the fight against cancer. Modern research strives to push forward the boundaries of our current understanding of this malignancy, but many efforts are still needed to find effective treatments for many types of cancer, as most of the drug discovery programs focus on a relatively small subset of druggable protein targets (such as kinases and G protein coupled receptors), thus excluding more than 85% of the genome [48]. Recent advances in large-scale genome sequencing and functional genomic studies have led to new opportunities for the development of innovative treatments. Novel therapeutic targets include structural proteins and transcription factors, as well as protein–protein interactions (PPIs). Their potential is now well established, but their modulation through small molecules is often difficult. The recent progress in peptide technology can meet the challenges of such a widening drug discovery landscape. Moreover, as already mentioned, peptides can achieve high target specificity and low toxicity, which are particularly important to develop safer and more effective anticancer therapies [48]. Importantly, cancer cells present the following physiological properties that distinguish them from the healthy ones: outside the membrane, the pH is more acidic (6.4 against physiological 7.4); the membrane is more fluid due to the higher concentration of cholesterol; and the surface of the cell presents an overall negative charge, unlike the neutrality of healthy cells. These properties can be exploited to overcome the lack of selectivity presented by traditional therapies [49].

**Figure 12:**
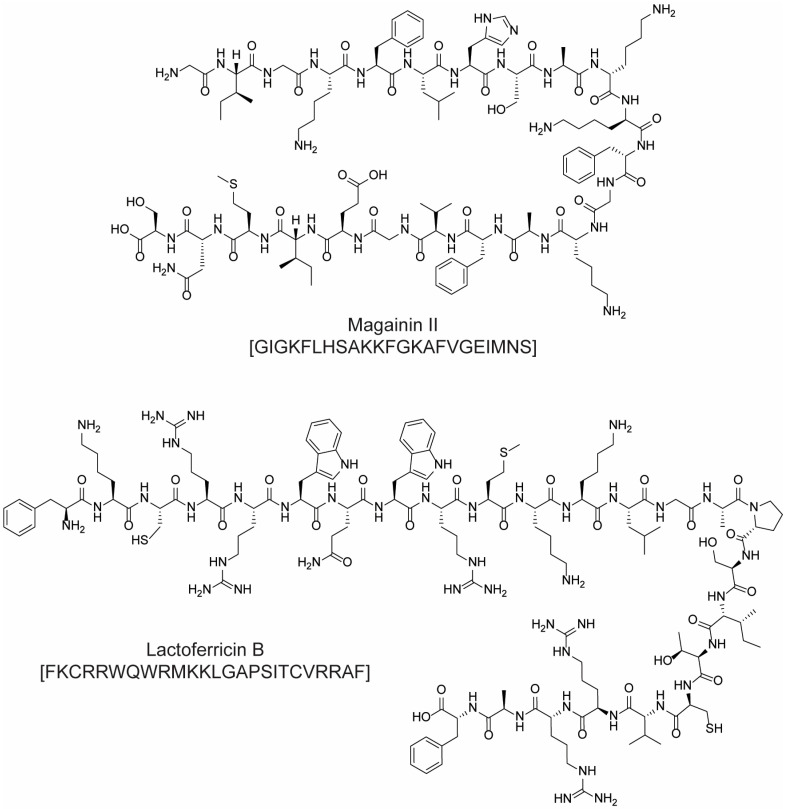
Chemical structures of magainin II and lactoferricin B. The amino acidic sequence (using the one letter code) is reported in squared brackets.

**Figure 13:**
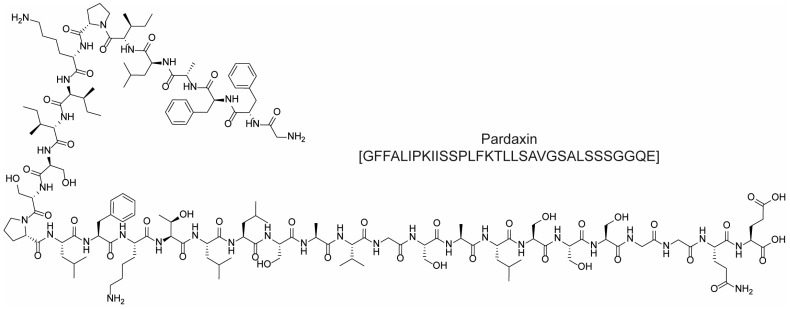
The chemical structure of pardaxin. The amino acidic sequence (using the one letter code) is reported in squared brackets.

**Figure 14:**
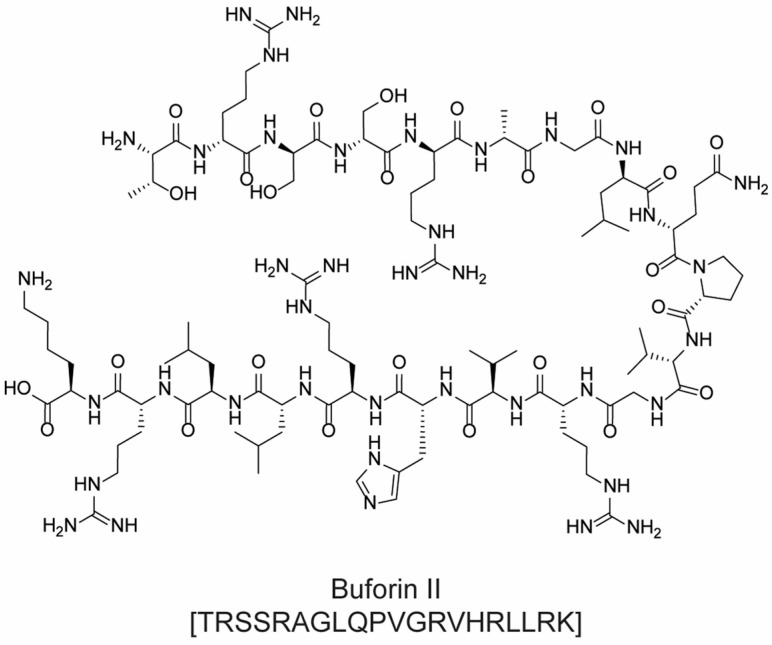
The chemical structure of buforin II. The amino acidic sequence (using the one letter code) is reported in squared brackets.

For example, cationic ACPs exploit electrostatic forces to interact directly with the outer membrane of cancer cells, which is more negatively charged than the surface of healthy cells. Accordingly, such peptides are mainly composed of basic and hydrophobic amino acids, which at a physiological pH, give an overall positive charge and amphipathic properties. Upon interaction with a biological membrane, these peptides can adopt different structures (i.e., α-helical, β-sheet, or extended), all of which have both a cationic face and a hydrophobic face. The amphipathic nature of such molecules allows their adhesion to the cell membrane and the subsequent translocation into the interior of the cells (in fact, they are sometimes referred to as “cell-penetrating peptides”). Their mechanism of action can be either direct or indirect: in the first case, cell death is caused by irreparable membrane damage and cell lysis, whereas in the second case, cancer cells are killed by intervention in cell death pathways, signal transduction pathways, and/or the cell cycle [48,53]. For example, magainin II (Figure 12) [54], lactoferricin B (Figure 12) [55], and the pleurocidin-family peptides NRC-3 and NRC-7 [56] act through a direct mechanism; pardaxin (Figure 13) [57] and buforin IIb (Figure 14) [58] induce apoptosis by different mechanisms.

Most cationic ACPs also have antimicrobial properties. Indeed, natural cationic peptides were firstly isolated from different organisms and identified as AMPs before their anticancer potential started to be investigated [59]. Today, extensive structure–activity relationship (SAR) studies and computational techniques enable the rational design of novel cationic ACPs [60,61,62]. Using machine learning, Shoombuatong et al. determined some common features among ACPs, such as a length of about 20–30 amino acids and a high abundance of lysine, glycine, and leucine [63]. Starting with this study, Hadianamrei et al. developed a rational design of anticancer peptides with a general formula C(XXYY)_3_, where C stands for cysteine, X represents isoleucine or leucine (hydrophobic amino acids), and Y is arginine or lysine (cationic amino acid) [64]. In their study, they tested a library of peptides presenting these features against cervical and colorectal cancer cells, and they observed that their compounds effectively entered the cells and caused apoptosis by damaging the mitochondrial membrane. The authors demonstrated that the selectivity was determined by the positive-negative interactions of the peptides and the membrane of tumoral cells. Their toxicity was instead correlated to the α-helix content, the hydrophobicity of the structure, and the surface activity, which is indicative of the amphiphilicity of the peptide. Moreover, the authors registered an increase in toxicity towards tumoral cells when a cysteine was added to the N-terminal and isoleucine residues were added to the C-terminal, while toxicity against healthy cells was always low [64].

Another class of peptides useful for cancer treatment is represented by “cell-targeting peptides” (CTPs), i.e., those that specifically bind to cell membrane proteins that are overexpressed in cancer cells, thus avoiding harm to normal healthy cells. Membrane proteins that are targeted by these agents include the epidermal growth factor receptor (EGFR), integrins, and G protein coupled receptors (GPCRs). The interaction of CTPs with these targets can result in the internalization of the peptide and/or modulation of the signaling from the receptor [48]. For example, the Arg-Gly-Asp (RGD) sequence is recognized and bound by integrins, and for this reason, it can be exploited for cell adhesion and internalization. Cilengitide (*vide infra*) and lunasin are two notable representatives of RGD-containing peptides. The latter is a natural peptide composed of 43 amino acids [SKWQHQQDSCRKQLQGVNLTPCEKHIMEKIQGRGDDDDDDDDD] originally isolated from soybeans, and it has well-characterized anticancer activity; it was demonstrated that lunasin inhibits histone H3 and H4 acetylation, induces apoptosis, and inhibits caspase-3 in vivo [65,66].

**Figure 15:**
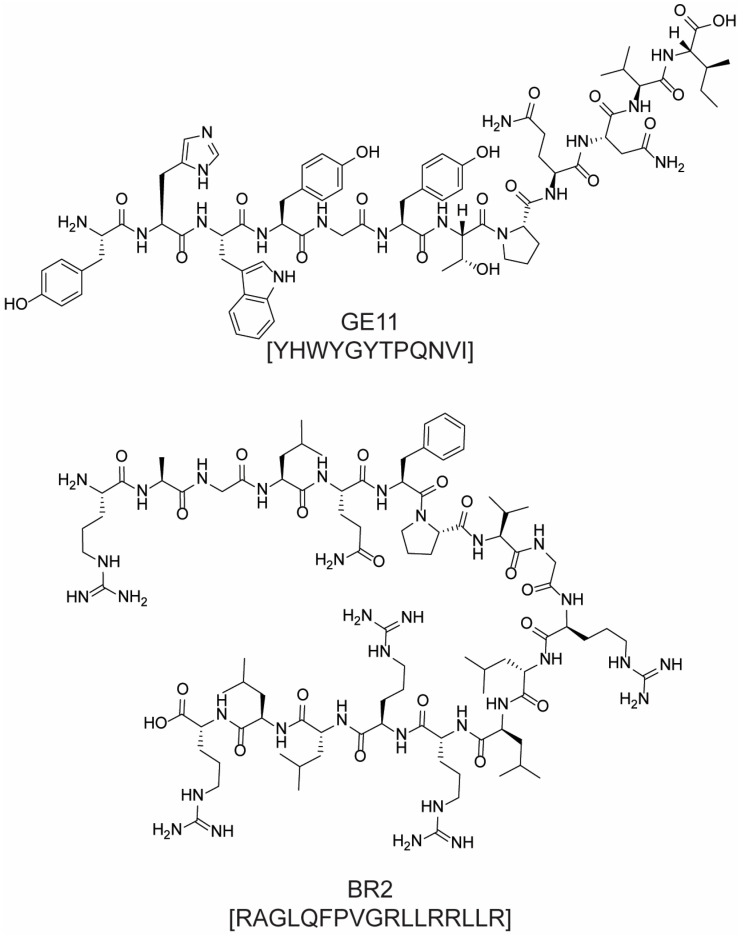
Chemical structures of the cell-targeting peptides GE11 and BR2. The amino acid sequence (using the one letter code) is reported in squared brackets.

Other CTPs may have poor or absent anticancer activity of their own, but they can be conjugated to chemotherapeutics for targeted drug delivery, thus improving treatment selectivity, efficacy, and safety. Examples include GE11 (YHWYGYTPQNVI, Figure 15)—a peptide with nanomolar affinity toward EGFR that has been conjugated to a wide range of anticancer agents [67,68,69]—and the 17-amino acid peptide BR2 (Figure 15), which was exploited to deliver a pro-apoptotic single-chain variable fragment (scFv) to colon cancer cells [70]. CTPs have also been used in combination with metal-based anticancer agents, an important class of chemotherapeutics developed since the serendipitous discovery of the antiproliferative activity of cis-platinum complexes [71]. In fact, the main limitation of these metal derivatives is their lack of selectivity, which entails many side effects such as nausea, anorexia, allergic reactions, and nephrotoxicity [72].

Recently, Boscutti et al. tested a series of Au(III)-peptidodithiocarbamato complexes whose peptide tail was varied in order to evaluate selectivity against tumoral cells overexpressing PEPT1 and PEPT2 receptors, i.e., specific proton-coupled transporters mediating the uptake of oligopeptides in different tissues. Some compounds showed an antiproliferative effect comparable to or even greater than the cis-platinum complex tested for reference [73].

Another recent application of peptides in cancer therapy is the stimulation of the immunogenic response. Many ACPs are in fact endowed with immunomodulatory properties that can be harnessed to effectively counteract tumor growth [48,53]. In 2021, Mauriello et al. probed the viability of immunizing C57BL/6 mice by administering tumor antigens (TuAs) or the corresponding heteroclitic peptides (hPep), i.e., variants of native peptides designed to stimulate stronger T cell responses [74]. Upon completion of the vaccination protocol, animals were implanted with tumor cell lines, and the specific anti-vaccine immune response, along with tumor growth, were evaluated. The immunization was very effective, resulting in a significant delay or suppression of tumor growth, even when implantation was performed two months after vaccination. These results indicated that tumor growth can be controlled by an established T cell memory specific for antigens structurally related with a TuA. This response was achieved by the authors exploiting synthetic hPep specifically designed from TuA sequences [74].

**Figure 16:**
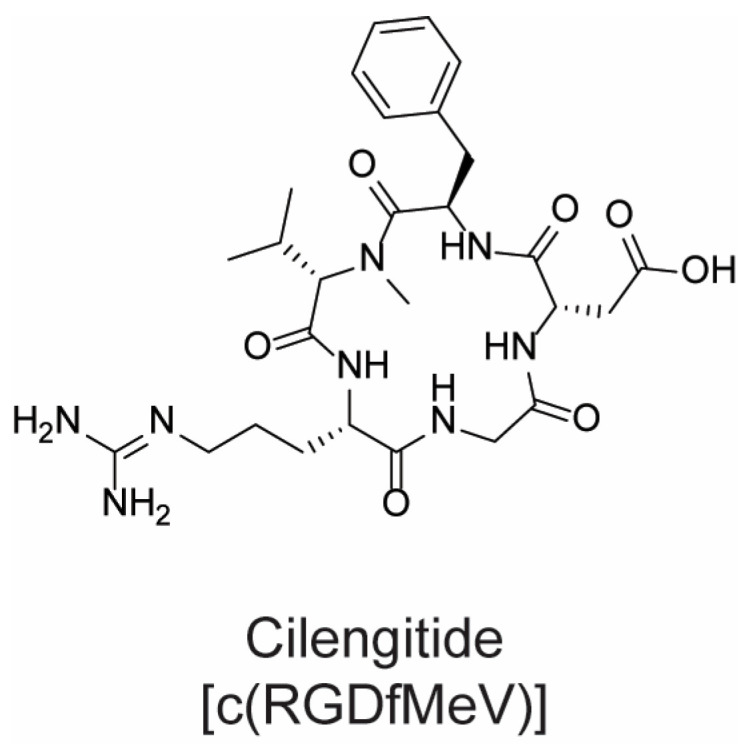
The chemical structure of cilengitide.

To summarize, peptides hold great promise in cancer treatment, despite their intrinsic limitations (such as fast enzymatic degradation and administration issues). As aforementioned, researchers have developed, over the years, different strategies to overcome such drawbacks. Peptide cyclization is a well-established structural modification that can be used to achieve this goal. By masking both the C- and N-terminal amino acids, the proteolytic degradation by peptidases is significantly hampered. Moreover, the constrained structure allows fewer conformations, thus favoring the binding to the target’s active site [48]. Particularly, in the field of anticancer therapy, cyclic peptides carrying the RGD sequence are one of the most known classes of peptidomimetic derivatives. These compounds are potent antagonists of αvβ3 and ανβ5 integrin subtypes, which are overexpressed in different cancer types, such as colon cancer, melanoma, breast cancer, and glioblastoma [75]. In the cyclic pentapeptide cilengitide [*c*(RGDfNMeV)] (Figure 16), the first integrin antagonist to reach clinical trials, the presence of dipeptide d-phenylalanyl–*N*-methyl valine allowed for fixing the RGD sequence into the correct bioactive conformation to bind in nanomolar concentration αvβ3 and αvβ5 integrin receptors [76].

Unfortunately, despite promising preliminary data, the use of cilengitide as an anticancer drug against glioblastoma has been discontinued due to failure in phase-III clinical trials, and consequently, the use of integrin antagonists has been set aside for a while [77]. Nevertheless, given their intrinsic very low toxicity towards healthy cells and their high affinity and selectivity for integrin overexpressed in various tumor forms, in the last years, RGD-based antagonists found a second life as drug delivery systems [78,79,80]. In this context, constrained dipeptide mimics, such as proline-derived azabicycloalkane amino acids, were used for the synthesis of *c*RGD antagonists [81,82], *c*RGD-cytotoxic bioconjugates [83], *c*RGD-based imaging probes [84,85], and *c*RGD-functionalized nanosystems [86,87].

### 2.4. Others

Obesity is a pathology characterized by a huge accumulation of fatty tissue in the body, whose consequences are extremely dangerous for health, without any real treatment approved yet. In the last decades, researchers have discovered that different peptides isolated from soybeans could be extremely useful in treating obesity due to their anorectic properties [88]. In recent years, Asokan’s group has shown that a tetrapeptide (ValHisValVal) derived from soybeans’ proteins is capable of stimulating lipolysis in apoptotic skeletal muscles caused by a full-fat diet. In addition, the ValHisValVal peptide is responsible for TNF-α regulation, whose level is elevated in people affected by obesity [89]. Additionally, LeuProTyrProArg, which is a peptide that comes from soybean glycinin, possesses anorectic activity; in fact, it is able to diminish serum glycerides and cholesterol without any impact on other body proteins [88]. The discovery of peptides capable of treating this pathology prompted the scientific community to develop peptidomimetic derivatives with improved biological activity and stability.

The peptide motif CKGGRAKDC has been investigated as a potential tool to treat obesity by selectively inducing apoptosis in the vasculature of adipose tissue. This sequence targets prohibitin, a multifunctional membrane protein that can be exploited as a vascular marker of adipose tissue [90]. Its derivative adipotide is a peptidomimetic with the sequence CKGGRAKDC-GG-_D_(KLAKLAK)_2_, which demonstrated a consistent reduction in white adipose tissue in three different species of monkey, causing a reversible functional change in the renal proximal tubule as a side effect. Its mechanism of action was elucidated; adipotide (also known as prohibitin-targeting peptide 1) binds prohibitin in the white adipose tissue vasculature, and the complex formed by ligand and receptor starts the apoptosis of white fat cells [91,92]. However, despite promising results in vivo, the first human clinical trial was discontinued in 2019 due to unspecified reasons [93].

**Figure 17:**
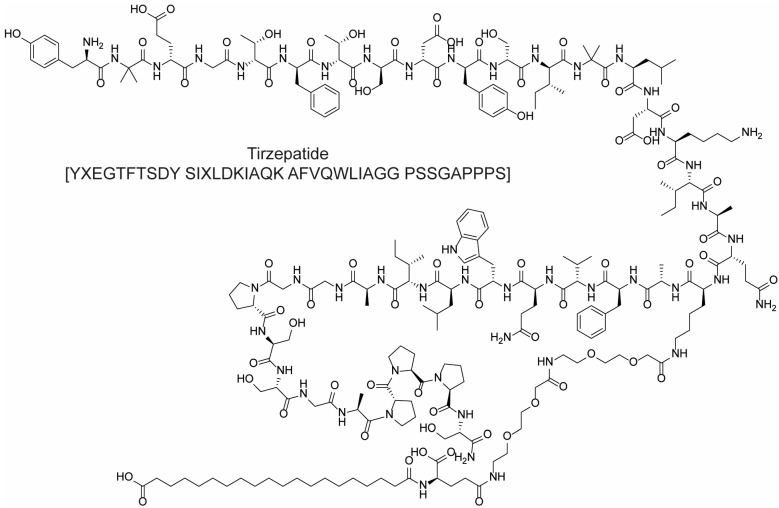
The chemical structure of tirzepatide. The amino acidic sequence (using the one letter code) is reported in squared brackets.

Tirzepatide (Figure 17) is a state-of-the-art investigational dual agonist for the glucose-dependent insulinotropic polypeptide (GIP) and glucagon-like peptide-1 (GLP-1) receptors, tailored specifically for treating type 2 diabetes. It acts by mimicking the physiological actions of both GIP and GLP-1, essential incretin hormones released from the intestines following nutrient intake, crucial for regulating blood glucose levels. Tirzepatide’s GLP-1 component enhances insulin secretion, reduces glucagon release, and slows gastric emptying, collectively mitigating postprandial glucose spikes and promoting glucose stability. Simultaneously, while the GIP component’s role in glucose regulation is complex, it works in tandem with GLP-1 to amplify the hypoglycemic effect. Importantly, tirzepatide has also shown potential in promoting weight loss, a crucial benefit for many type 2 diabetes patients who also struggle with obesity. Since September 2021, comprehensive clinical trials were in progress to determine tirzepatide’s safety and effectiveness, with results likely to shape its potential approval and clinical adoption. Owing to its dual-action mechanism, tirzepatide might surpass other GLP-1 receptor agonists in delivering both glycemic control and weight loss advantages.

Another therapeutic field where peptides find application is that of cardiovascular diseases (CVDs), which are the leading cause of death worldwide, with an estimated 17.9 million deaths per year [94]. Therefore, the development of novel and effective therapies for CVDs is an urgent need, as it is estimated that between 1990 and 2013 the number of deaths caused by these pathologies increased by about 40% due to population growth and aging [95].

**Figure 18:**
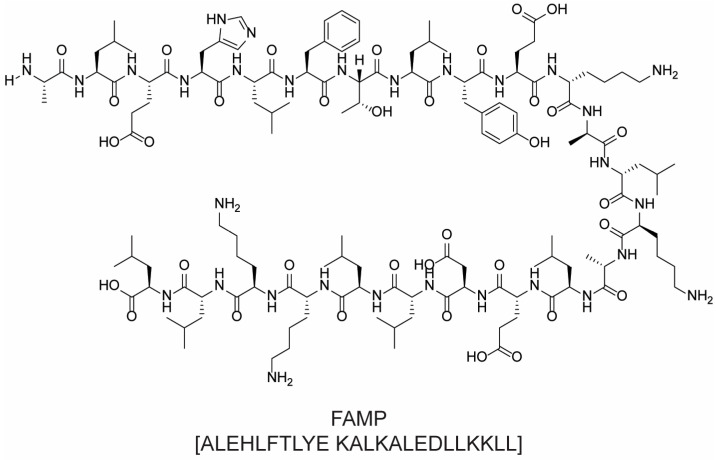
The chemical structure and aminoacidic sequence of the novel ApoA-I mimetic peptide, FAMP.

High levels of low-density lipoprotein (LDL) cholesterol are one of the major causes that leads to an increase of CVD episodes. At the same time, high levels of high-density lipoprotein (HDL) result in the removal of free cholesterol and its consequent transport to the liver for clearance. It is well known that apolipoprotein AI (ApoA-I) constitutes the major protein-component of HDL cholesterol; this consideration led to the investigation of it in depth for its anti-atherogenic activity [96]. The structure of this peptide is constituted by 10 amphipathic α,α-helices, which have been used as a template in drug discovery programs to obtain different molecules that mimic ApoA-I activity [97,98]. For example, ApoA-I has been correlated with Tangier disease, a serious condition that consists of a severe HDL deficiency [99]. This pathology can be induced in mice by the mutation of ABCA1 (ATP-binding cassette transporter); on the other hand, ABCA1 overexpression in transgenic mice leads to an increment of ApoA-I cholesterol efflux [100,101]. This indicates that there is a correlation between ABCA1 and the regulation of plasma HDL-cholesterol levels. The viability of ApoA-I as a template for the development of peptidomimetic therapeutics has been corroborated by the observation that FAMP (Fukuoka University ApoA-I mimetic peptide) increases the biological function of HDL and exerts atheroprotective effects at the same time, leading to a reduction of aortic plaques of about 50% in ApoE knockout mice on a high-fat-diet [102]. Notably, while ApoA-I is constituted by 243 amino acids, FAMP structure consists of only 24 amino acids, with an amphipathic helical structure that is essential for association with lipids [103]. While several other ApoA-I mimetic peptides (like D-4F, L-4F, and L37pA) are highly lipid-specific, consequently allowing cholesterol efflux through non-specific ways, FAMP (Figure 18) is designed to specifically interact with ABCA1, thus avoiding the non-specific lipid efflux [104]. The first in vivo demonstration of FAMP activity was performed by Takata and coworkers in 2016. This research project gave new insights about FAMP, underlining its favorable effect on angiogenesis and the capability of improving functional recovery in hindlimb ischemia in mice [103].

## 3. Peptide Synthesis: Challenges and Opportunities

As previously highlighted, peptides are pivotal compounds in both therapy and drug discovery, anticipated to significantly influence the future trajectory of the pharmaceutical industry. Nonetheless, the synthesis of peptides is often characterized as one of the most wasteful and environmentally unfriendly chemical processes [21,105,106,107]. Addressing this important drawback is crucial for the achievement of widely available therapeutic tools. In fact, in 2018, the ACS Green Chemistry Institute^®^ Pharmaceutical Roundtable (GCIPR) included methodologies for catalytic/sustainable (direct) amide or peptide formation in their list of the “10 green areas of research” [108].

Since each amino acid presents (at least) two reactive moieties that can be involved in the formation of an amide bond, the synthesis of peptides has always represented a challenge. Both protective groups and activating groups are needed to avoid side-reactions and to yield the new peptide bond in a chemoselective fashion. Furthermore, the potential racemization represents an important issue to be considered in the choice of reaction condition. Peptide chemical synthesis is traditionally set from C to N terminus (contrary to what happens during biosynthetic processes); therefore, the carboxylic group needs to be activated, while the amino group must be protected. The most widely used amino-protecting groups are fluorenylmethyloxycarbonyl (Fmoc), tert-butyloxycarbonyl (Boc), or benzyloxycarbonyl (Cbz), while common activating reagents can be classified as carbodiimides, active esters, phosphonium salts, and uronium/aminium salts. Finally, it must be noted that each coupling reaction might give the incomplete conversion of the starting materials, and the remaining reactants might take part in the next coupling step, giving a large number of side-products. For these reasons, peptide synthesis is a laborious process that involves several steps of protection/deprotection, activation, and purification. Over the years, significant efforts have been made to improve efficiency and, more recently, environmental sustainability, which is discussed in the following sections.

A crucial consideration in peptide preparation is the selection of the synthetic strategy. One can elongate the chain by adding amino acids sequentially (step-by-step approach) or by merging oligopeptides (fragment condensation approach). The former is more time-consuming but minimizes racemization risks, whereas the latter facilitates the simultaneous production of multiple fragments that can subsequently be combined. Beyond the time efficiency, the fragment condensation approach typically affords higher overall yields compared to the step-by-step method. This is because the overall yield is determined by multiplying the yield of each individual step [109]. For instance, for an 8-amino acid peptide, the overall yield exploiting an 80% step-by-step approach would be 21% (0.8^7 × 100). In contrast, condensing two 4-amino acid fragments and assuming they were obtained with the same yields, the overall yield for the final product will result in 41% (0.8^4 × 100, considering three condensation steps for each fragment and the final coupling between the two tetrapeptides). Due to these advantages, the fragment approach is generally favored for chains exceeding four or five residues.

Although the fragment-based method is a very powerful method, it suffers from some drawbacks that prevent it from being the suitable method for the synthesis of very long polypeptides or proteins, e.g., the high probability of racemization occurring during the condensation steps. Through a technique called “Native Chemical Ligation” (NCL), it is possible to partially overcome the limitations of the classic peptide synthesis approach. This approach is based on the possibility of bonding two unprotected peptide segments (up to 100 amino acids long) endowed with a C-terminal thioester group and an N-terminal cysteine residue [109]. The cysteine’s thiol moiety of one chain can react with the thioester group of the other, affording a transient intermediate that, after a spontaneous rearrangement, gives rise to a native peptide bond between the two chains. This approach made accessible a variety of long peptides that previously represented a great synthetic challenge. Moreover, the necessity of having an N-terminal cysteine residue has been overcome over the years by the development of different strategies. One of these exploit homocysteine residues. After the ligation, it is possible to perform S-methylation at the homocysteine site; therefore, NCL can also be considered a strategy to create polypeptides with methionine junctions [110]. Other techniques involve the synthesis of thiolated building blocks or the auxiliary-mediated ligation (AML) that consists of the introduction of a lateral thiolated chain to the N-terminus of the polypeptide that can easily be removed after the ligation reaction under different conditions (photolysis or acidolysis) [111]. The development of desulphurization techniques has extended the NCL to alanine junctions; it is possible indeed to remove the thiol group after the ligation by using Pd or other metals. However, some drawbacks of this strategy, such as the environmental impact of the process, the need to protect native cysteines when performing the desulfurization, and the slow rate of some NCS, have prompted the search for alternative reactions involving, for example, the use of selenium. Overall, this convergent approach offers many advantages, such as potentially higher yields and crude purities, and the consumption of lower amounts of solvents, often water [21].

Besides the overall synthetic strategy (step-by-step vs. fragment condensation), different approaches can be adopted in the formation of the single amide bond. The most relevant today, in terms of efficacy and industrial applicability, include solid- and liquid-phase peptide syntheses and microwave-assisted and chemoenzymatic peptide syntheses. These are briefly described in the following, as we discuss their pros and cons, especially in relation to sustainability and environmental impact.

### 3.1. Solid-Phase Peptide Synthesis (SPPS)

**Figure 19:**
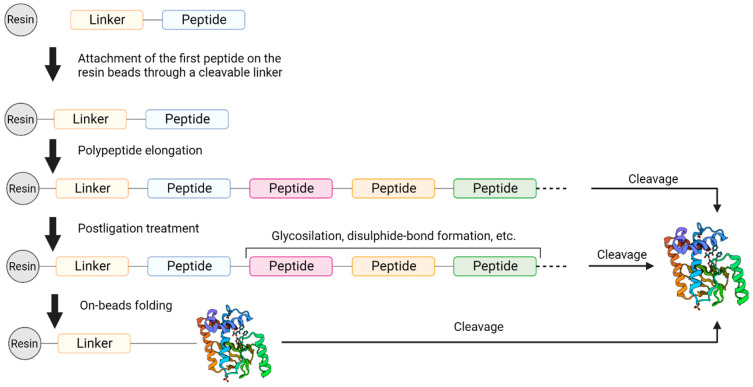
A schematic representation of solid-phase peptide synthesis. Picture adapted from reference [113].

In 1963, Bruce Merryfield developed an innovative way to obtain peptides, known as solid-phase peptide synthesis (SPPS), by using a resin as solid support for synthesis. The first amino acid of the chain is chemically bound to the resin through the use of a linker, and the free amino group can react with a second amino acid that has its amino group protected but the carboxylic moiety free and available for activation. After that, the protecting group is removed, and a new amino acid is added. This iterative process has been used to build peptide chains of up to 100 amino acids, and it quickly became the benchmark technology in peptide production, although it must be noted that the synthesis of peptides with more than 50 amino acids is typically challenging, with final yields unavoidably diminishing as the number of coupling steps increases. To limit the formation of side-products derived by incomplete couplings, a capping step is often applied in which the unreacted free amine is acetylated by a reaction with Ac_2_O. The last steps of the sequence are the detachment of the peptide from the resin and the final purification. This is particularly crucial, since several side products (e.g., truncated and capped peptides) can be obtained during the process [112,113]. A schematic representation of the whole synthetic process is shown in Figure 19.

Bruce Merryfield developed his synthetic strategy using a chloromethylated polystyrene resin (also called Merrifield resin), and since then, many resins have been developed that can be divided into three main classes: polystyrene resins (PS), PS-functionalized polyethylene glycol (PEG) resins, and pure cross-linked PEG resins [113,114].

The SPPS presents many advantages. Above all, the operations can be automated, and the time can be reduced with respect to classical solution-phase synthesis, permitting an easier production of different peptides at the same time. For these reasons, even if this procedure is more expensive than the traditional one, nowadays, SPPS is widely used for peptide synthesis. The main disadvantage of SPPS, from a “green” perspective, is the huge volume of organic solvents required; N,N’-dimethylformamide (DMF), dichloromethane (DCM), and N-methyl-2-pyrrolidone (NMP) are the most widely used, followed by diethyl ether and tert-butyl methyl ether. These traditional solvents, especially DMF and NMP, are toxic for reproduction and have consequently been classified as two of the six substances of highest concern by the European Regulation for Registration, Evaluation, Authorisation, and Restriction of Chemicals (REACH) [115]. Greener alternatives are needed; therefore, extensive efforts have been made to introduce new methods to either replace toxic solvents or to minimize their impact (e.g., by recovery or by diminishing the volume needed).

#### 3.1.1. Green Solvents

The use of safer alternatives (i.e., green solvents) to the aforementioned traditional organic solvents represents a promising strategy for improving the sustainability of therapeutic peptide production.

**Figure 20:**
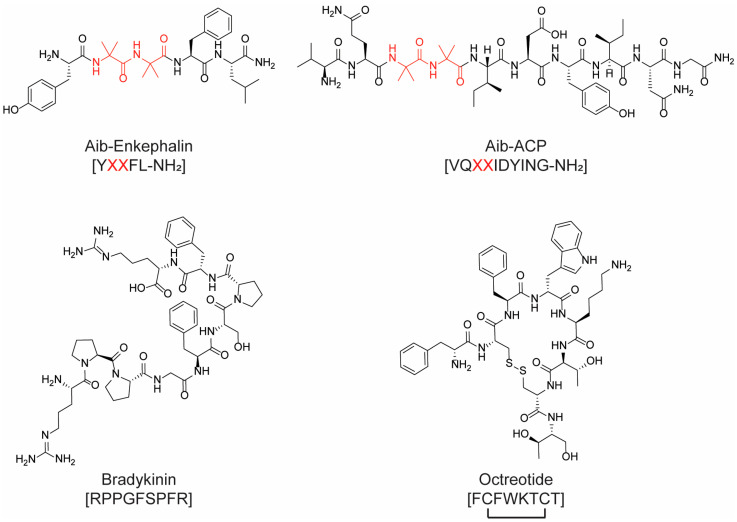
Chemical structures of some of the peptides synthesized in the literature using greener solvents. X = 2-Aminoisobutyric acid (Aib), highlighted in red in the structures.

Albericio and coworkers proposed the use of acetonitrile (ACN) and tetrahydrofuran (THF) as alternatives to DMF and NMP in SPPS, in combination with PEG-based resins [116]. The same group reported the synthesis of the Aib-enkephalin pentapeptide (H-Tyr-Aib-Aib-Phe-Leu-NH_2_) (Figure 20) using greener solvents such as 2-methyltetrahydrofuran (2-MeTHF), which can be derived from renewable resources [117], and cyclopentyl methyl ether (CPME) as environmentally friendlier alternatives to DMF [118]. Additionally, they reported the synthesis of the decapeptide Aib-ACP (H-Val-Gln-Aib-Aib-Ile-Asp-Tyr-Ile-Asn-Gly-NH_2_) (Figure 20), replacing DMF with THF and ACN and exploiting SPPS methodology on the polyethylene glycol resin ChemMatrix. Both THF and ACN showed a better coupling efficiency than DMF. Moreover, the use of these solvents allowed for the suppression of the formation of by-products [119]. Afterwards, the use of N-formylmorpholine (NFM), isosorbide dimethyl ether, dimethyl carbonate (DMC), and γ-valerolactone (GVL) has been deeply investigated. GVL can replace DMF in Fmoc removal, working with both PS or ChemMatrix resins, while NFM showed excellent performance only when the ChemMatrix polymeric support was used during the synthesis [120]. Lawrenson et al. investigated the use of cyclic carbonates, in particular propylene carbonate (PC) and ethylene carbonate (EC), as solvents for both coupling and deprotection steps using a range of common protecting groups and coupling agents [107]. PC can replace DMF during all steps of the synthesis in solution and SPPS, using either the Fmoc or Boc strategy with the ChemMatrix resin. In addition, the use of PC in place of DMF in the SPPS of the nonapeptide bradykinin (H-Arg-Pro-Pro-Gly-Phe-Ser-Pro-Phe-Arg-OH) (Figure 20) allowed for an easier purification of the final product.

Scientists at Novartis screened a series of solvents in the synthesis of the linear precursor of cyclic peptide octreotide (Figure 20), used in the treatment of acromegaly and gigantism [121]. Among the 34 screened solvents, N-butyl-2-pyrrolidinone (NBP) turned out to perform well in all synthetic steps and was successfully used for the preparation of the target molecule. After the successful validation of NBP in SPPS processes, Martelli et al. explored the class of N-alkylpyrrolidones in “green” coupling reactions [122]. After a careful investigation of reaction parameters such as resin swelling, the solubility of starting materials and intermediates, the removal of the Fmoc protecting group, and coupling efficiency, N-octyl-pyrrolidone (NOP) emerged as the best candidate.

Another attractive possibility is the replacement of hazardous solvents with water. Hojo et al. proposed an environmentally friendly approach based on water-dispersible amino acid nanoparticles, proving its utility with the successful solid-phase synthesis of Leu-enkephalin amide in water [123]. The employment of the water-dispersed nanoparticulate reactants provides the advantage of an easier separation of the excess reactants from the resin by filtering through a microfilter. Afterwards, the same group reported an aqueous microwave peptide synthesis, which showed promising applicability in the preparation of racemization-sensitive derivatives, such as histidine-containing peptides [124]. A comprehensive work summarizing all the developments undertaken by Hojo’s team in this regard was recently published [125]. Lipshutz and co-workers reported a general method for peptide bond formation in an aqueous micellar medium at room temperature, in which the reaction medium can be easily recycled [126]. In particular, the surfactant dl-α-Tocopherol methoxypolyethylene glycol succinate, known as TPGS-750-M, was used to spontaneously assemble water-insoluble reagents into nanomicelles. Upon dissolution in water, the micellar core, composed of racemic vitamin E, easily accommodates the organic substrates (amino acid/peptide partners) and coupling agents; these unique arrangements in small micelles (50−60 nm) act as “nanoreactor” thanks to the associated hydrophobic effect that tends to accelerate reactions and enables transformations to occur typically under milder conditions [127]. This approach successfully addresses several environmental concerns, including replacing organic solvents with extremely small amounts of water as the reaction medium, avoiding the use of benzotriazole activators, reusing aqueous surfactant solutions, and using minimal workup/purification organic solvents. This methodology may be employed for the synthesis of small peptides, mainly tri- and tetrapeptides. Similarly, in 2020, Handa and co-workers reported the use of amphiphile PS-750-M, a surfactant characterized by the presence of a tertiary amide bond involving the l-proline moiety, which in fact mimics solvents such as DMF, dimethylacetamide, and NMP [128]. The resulting micellar system allowed for fast coupling reactions in water by using 1-ethyl-3-(3-(dimethylamino)propyl)carbodiimide (EDC) without hydroxybenzotriazole, rather than expensive and specialized coupling agents. Notably, all the reactions were reproducible on the gram scale, and no column chromatography, extraction, or recrystallization were required for obtaining the pure products, which were collected by simple filtration. Under basic aqueous conditions, no traces of Fmoc deprotection were observed. Finally, although working in the absence of benzotriazole derivatives, the authors did not evidence the formation of any epimerization product.

In recent years, the use of green binary solvent mixtures in SPPS represents a way forward, opening a revolutionary possibility for new solvents to efficiently accomplish all steps of the peptide synthesis. Indeed, the appropriate polarity and/or viscosity for successful swelling of the resin and reagent solubilization are more frequently satisfied by using a binary mixture than with a single solvent system. Pawlas and Rasmussen used ethyl acetate (EtOAc) and dimethylsulfoxide (DMSO) as solvents. They synthesized a crude model 6-mer peptide (Fmoc-Cys(H)_6_-NH_2_) via SPPS on PS/DVB resins in a higher yield and purity in DMSO/EtOAc (1:9 ratio) rather than in DMF [106]. In addition, both DMSO and EtOAc were recycled by distillation (isolated in 70% and 86% yield, respectively) for further synthetic uses. The coupling agent ethyl (hydroxyimino)cyanoacetate (Oxyma) of the waste stream from the SPPS process was also recovered and used together with the recycled solvents in the SPPS coupling. The obtained crude peptides were indistinguishable from those obtained by using the corresponding “virgin” starting materials, thereby proving that the recycled chemicals can be reused without detrimental effects on the synthesis.

At the same time, the application of green solvent mixtures as combinations of cyrene (dihydrolevoglucosenone), sulfolane, or anisole with dimethyl carbonate (DMC) or diethyl carbonate (DEC), in different proportions, was explored [129]. In particular, three binary mixtures (DEC/cyrene in 7:3 ratio, DEC/sulfolane in 7:3 ratio, and DMC/anisole 3:7 ratio) were explored in a full SPPS protocol for the synthesis of the more challenging sequence Aib-ACP. The mixture anisole/dimethyl carbonate (7:3) on ChemMatrix-RinkAmide gave the best results in terms of HPLC purity. Very recently, a systematic investigation was conducted on how the composition of green binary solvent mixtures affects Fmoc removal, peptide coupling, and common side reactions in SPPS [130]. It was further demonstrated that altering the composition of these binary solvent mixtures during synthesis offers a straightforward method to reduce certain side reactions in SPPS, such as the inhibition of Arg-lactamation and aspartimide formation.

#### 3.1.2. Green Coupling Agents

Currently, in the realm of peptide synthesis, most amide coupling methods rely heavily on the utilization of benzotriazole derivatives. These reagents are well-regarded for their ability to facilitate efficient coupling reactions, minimize amino acid racemization, and maintain stability in solid form. Notably, certain derivatives, such as HATU, HCTU, and TBTU, based on uronium chemistry, even exhibit stability in the solution. Nevertheless, it is worth noting that the benzotriazole structure has been reported to have explosive properties, which can pose challenges during scale-up processes or when working at elevated temperatures [128]. Furthermore, these reagents are associated with poor atom efficiency. Remarkable reports from Albericio’s lab paved the way for future development on the coupling reagents and racemization suppressors [131].

**Figure 21:**
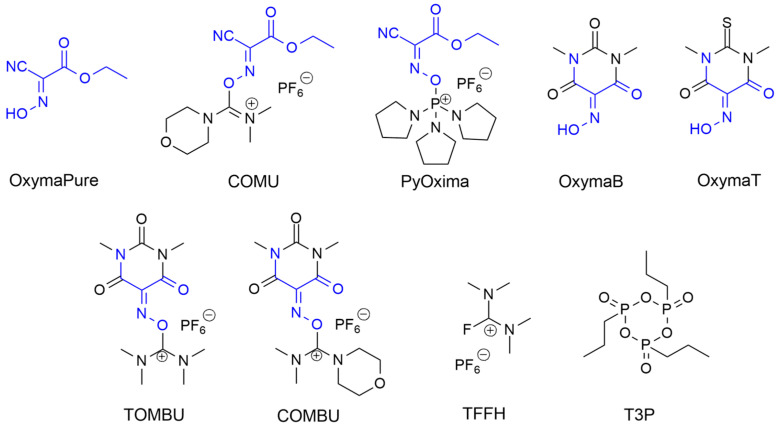
Greener coupling reagents. The Oxyma structure is highlighted in blue.

The ethyl cyano(hydroxyimino)acetate, known as OxymaPure^®^ (Figure 21), hereafter just Oxyma, and the corresponding potassium salt K-Oxyma were first proposed in 2009 by Albericio’s team as effective epimerization-suppressing additives [132]. These new peptide-coupling reagents also represent an attractive alternative to benzotriazoles, due to their compatibility with greener solvents and an improved balance between reactivity, solubility, and stability in various solvents. Moreover, Albericio’s group showed the high performance of COMU (Figure 21), a uronium-type coupling reagent based on the Oxyma scaffold, in microwave-assisted SPPS. The advantages of COMU over classic benzotriazole-based reagents (HATU, HBTU, HCTU, TBTU) were proven in terms of solubility and (often) coupling efficiency; moreover, the inclusion of the dimethylmorpholino skeleton in COMU resulted in a 50% higher solubility in DMF than HOTU, a uronium-modified Oxyma derivative [131]. COMU was also used for amide bond formation and for the synthesis of dipeptides in the presence of TPGS-750-M, a designed surfactant that when dissolved in water assembles into nanomicelles [126]. The authors reported that by working under environmentally benign conditions, e.g., using water as a solvent, it was possible to increase the E-factor and the recyclability of the reaction medium. Additionally, coupling performed in the presence of COMU led to the obtainment of desired products with no or negligible racemization. Besides COMU, many other Oxyma derivatives were introduced in ordinary laboratory practices in recent years, with several structure modifications, such as PyOxyma, Oxyma B, Oxyma T, TOMBU, and COMBU, all shown in Figure 21. However, these coupling additives did not possess a stronger applicability to green chemistry than the precursors OxymaPure and COMU. Finally, halogenformamidinium salts were also considered as a class of green coupling agents, which includes tetramethylfluoroformamidinium hexafluorophosphate (TFFH) and the most recent cyclic propylphosphonic anhydride (T3P^®^) (Figure 21) [21].

**Figure 22:**
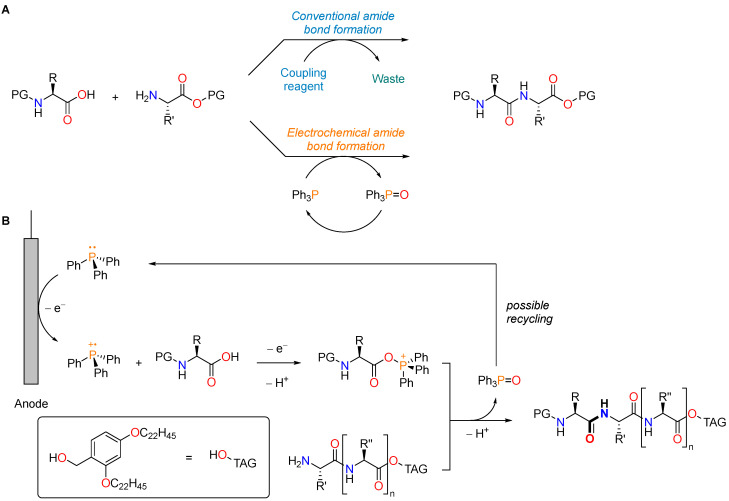
(**A**) A schematic comparison between the conventional peptide bond formation and the electrochemical approach. (**B**) Details of the mechanism of electrochemical peptide synthesis by the anodic oxidation of Ph_3_P.

However, the common drawback of all these coupling reagents is the fact that they are needed in stoichiometric quantities, which leads to the production of large amounts of waste. Current research therefore aims at innovative approaches to amide bond formation, with improved efficiency and sustainability. In 2007, the American Chemical Society Green Chemistry Institute Pharmaceutical Roundtable (ACS GCIPR) voted, as one of the key green chemistry research areas, the “amide formation avoiding reagents with poor atom economy”, later refined as “general methods for catalytic/sustainable (direct) amide or peptide formation” [108]. An in-depth discussion of advances and opportunities in this field is beyond the scope of the present work and has been reviewed in other excellent publications [133,134,135]. However, it is worth mentioning that a recent example of greener alternatives for peptide bond formation was disclosed by Nagahara and coworkers for the preparation of the commercial oligopeptide API leuproprelin [136]. In this work, the authors developed an electrochemical peptide synthesis in a biphasic system and exploited a potentially recyclable reagent, thus limiting the amount of waste and offering an alternative to previously reported catalytic methods. In detail, triphenylphosphine (Ph_3_P) was used as a coupling reagent precursor. The active species is an electrophilic phosphine radical cation, generated by the anodic oxidation of Ph_3_P, which activates carboxylic acid (Figure 22). Upon amide bond formation, triphenylphosphine oxide (Ph_3_P=O) is produced as a stoichiometric byproduct. However, contrary to the waste produced by “traditional” coupling reagents, in this case, the byproduct can be recycled and reused. Different methods, both chemical and electrochemical, have been reported for the large-scale reduction of Ph_3_P=O to Ph_3_P [137,138,139]. To facilitate the isolation of the desired product and the recovery of Ph_3_P=O, a biphasic system (ACN/*c*-Hex) was employed. The carboxylic acid was protected with a hydrophobic tag that makes the growing peptide soluble in *c*-Hex, thus enabling a liquid-phase peptide synthesis (LPPS, *vide infra*) approach, whereas the other reagents and byproducts are dissolved in ACN. This system demonstrated a broad substrate scope, with applicability toward both Fmoc- and Boc-protected amino acids, high reaction yields, and avoiding epimerization. The viability of this approach was further confirmed with the synthesis of leuprorelin in a 45% yield over 16 steps.

### 3.2. Microwave-Assisted Peptide Synthesis

Microwave (mw) irradiation has been used for decades in organic synthesis as a powerful way to (i) optimize heat transfer, (ii) reduce reaction time, (iii) obtain cleaner crudes, and (iv) enhance reactivity and/or solubility in alternative solvents. Altogether, these features make mw irradiation a versatile, greener, and more efficient option compared to traditional heating (e.g., oil baths and mantles), with broad applicability in organic synthesis and drug discovery [140]. The advantage of mw-assisted organic synthesis is based on the higher efficiency of dielectric heating compared to conventional heat transfer. This can reduce reaction times of an order of magnitude, speeding up the synthetic process, which is particularly advantageous in production programs. The efficacy of dielectric heating is strictly correlated with the ability of the solvent or reagents to absorb mw energy; therefore, polar solvents represent the best medium to perform reactions under mw irradiation. Not only DMF (which is one of the most popular solvents used to perform amide couplings) meets this requirement, but also many other greener alternatives, including alcohols, water, DMSO, ACN, and acetone [141].

Accordingly, mw-irradiation has been successfully exploited for peptide synthesis, enabling rapid and efficient couplings even with problematic substrates, while suppressing the typical side reactions of high-temperature peptide synthesis (e.g., aspartimide formation, cysteine epimerization, γ-lactam formation in arginine) [142]. Furthermore, the versatility of mw-assisted peptide synthesis allows for integration with other green approaches, such as alternative solvents and coupling agents described in the previous sections. The possibility of obtaining high-purity crude products with short reaction times prompted the development of dedicated automated instrumentation, such as the peptide synthesizers produced by CEM and CSBio corporations. Recently, these leading companies in the field of mw applications have also addressed the main drawback that has always been associated with mw-assisted organic synthesis, which is its limited scalability. In fact, this approach was traditionally regarded as more suitable for bench-scale reactions and early drug discovery rather than the industrial-scale production of APIs. However, this is changing with the recent introduction of batch-scale reactors, suitable for the GMP manufacturing of peptides in the (multi-) kilogram scale.

The advantages of mw-assisted peptide synthesis have been demonstrated by several researchers. For example, Collins et al. proved that, with proper reaction optimization, it is possible to increase product purity and yield while significantly reducing reaction time and total waste production (up to 90% compared to standard SPPS protocols) [143]. The authors supported the broad applicability of their mw-based protocol with six known peptides from 10 to 42 amino acid length, involving different synthetic challenges. The products’ purity was increased from the 42–90% range to 61–93% (depending on the target peptide), and the coupling time was reduced from a maximum of 60 min to a standard cycle of 4 min. Analogously, Vanegas Murcia and coworkers compared the synthesis of an 82-residue-long chimeric peptide under conventional and mw-assisted SPPS. Despite some synthetic difficulties related to the length and amino acid sequence, the total reaction time was shortened (from 162 h to 38 h), while crude yield and percentage recovery after purification were enhanced (from 9% to 13%) when employing mw-irradiation [144].

As aforementioned, mw-assisted peptide synthesis can be easily integrated with other green approaches, further reducing the environmental impact of the whole process. For example, Albericio and co-workers developed a green SPPS protocol using mw-irradiation in water, thus avoiding the use of toxic organic solvents such as DMF and NMP. After screening several coupling reagents and solid supports, the best results were obtained with EDC in combination with HONB (N-hydroxy-5-norbornene-endo-2,3-dicarboxyimide) on Rink Amide TentaGel™ using Boc-protected amino acids [145]. Similarly, Mahindra et al. reported an environmentally friendly, mw-assisted protocol for solution-phase peptide synthesis (i.e., without resins). The authors used TBTU/HOBt/DIEA as a coupling mixture in water. The use of mw enables mild reaction conditions, short reaction times, lower amounts of reactants, and compatibility with both N-Boc- and N-Fmoc-protected amino acids, giving the desired product in high yield and purity without racemization [146]. More recently, Schütznerová and coworkers focused on the Fmoc removal step in SPPS, seeking a greener alternative to the generally used and hazardous piperidine in DMF. The authors exploited anisole as a solvent and safer bases such as morpholine, DBU, and NaOH. Furthermore, it was demonstrated that the reaction time can be reduced, without compromising yield and purity, by exploiting mw-irradiation. The applicability of these green protocols was tested on model pentapeptides Leu-enkephalin and Aib-enkephalin [147].

### 3.3. Liquid-Phase Peptide Synthesis (LPPS)

As discussed before, Merrifield’s development of SPPS represented a breakthrough in peptide synthesis. However, with its increasing popularity, its limitations became more evident as well. These include the large excess of reagents and solvents needed for high conversion and purification and the aforementioned side reactions. Accordingly, significant efforts have been directed toward the identification of alternative technologies endowed with the potential to overcome such drawbacks. Among these, the most popular is liquid-phase peptide synthesis (LPPS), sometimes referred to as peptide-anchored LPPS (PA-LPPS) or tag-assisted LPPS to underline the difference with classical solution peptide synthesis (CSPS) [148]. LPPS can be regarded as a hybrid between SPPS and CSPS, aimed at combining the advantages of both techniques and minimizing their weak points. The amide coupling is performed in the solution, and the growing peptide chain is supported on a soluble “tag” or “anchor”. The kinetics of homogeneous solutions can effectively limit the amount of reagents to almost stoichiometric quantities, thus avoiding the large molar excesses typical of SPPS. Similarly, the volume of solvents needed in LPPS is significantly less than in SPPS, in compliance with green chemistry’s principles. This is strictly dependent on the characteristics of the tag, which must be lipophilic enough to ensure that the growing peptide is soluble in an organic layer, unlike the reagents and byproducts, thereby facilitating the work-up (e.g., by simple precipitation or extraction). From a structural point of view, the main difference between LPPS and SPPS supports is that, while the former is a small, well-defined molecule, or soluble polymer, the latter is a high-molecular-weight solid polymer. Accordingly, in LPPS there is no need for resin swelling and washing, making the solvent consumption even orders of magnitude lower than in SPPS [105,148].

**Figure 23:**
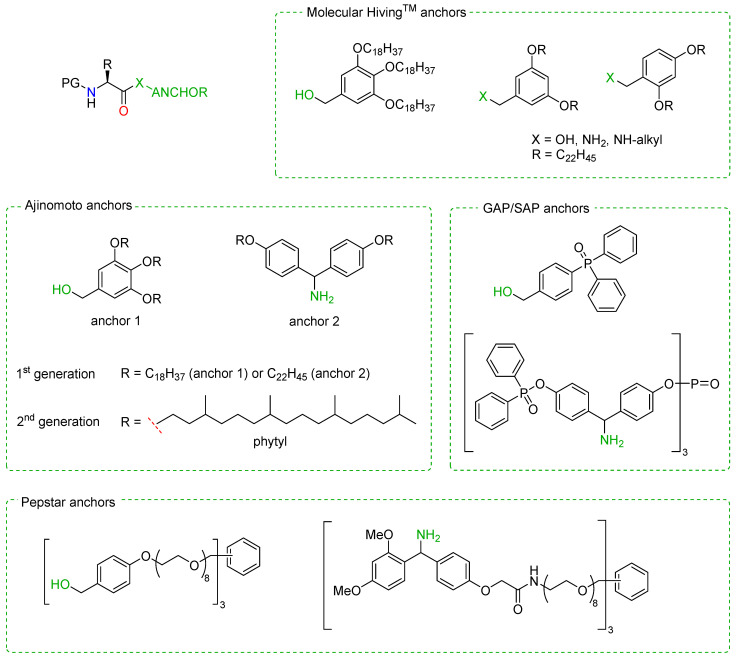
Examples of anchors used in LPPS. Picture adapted from reference [105].

The first soluble tags developed between the 1970s and 1980s were based on polyethylene glycol (PEG) chains, whereas over the last 20 years, there has been an outburst of innovation in this field, encompassing perfluoroalkyl, PolyCarbon, and phosphorus-containing tags, as well as hydrophobic polymers and ionic liquids. An in-depth description of these categories is beyond the scope of this review and has been excellently covered in the recent literature [148]. A few relevant examples are reported in Figure 23.

The advantages of LPPS also include the applicability of automation and direct monitoring (e.g., by HPLC) and the possibility to achieve the large-scale production of peptides using common reagents and standard, multi-purpose industrial plants [105,149]. Hence, LPPS is a valid alternative to SPPS, with its own advantages and downsides that must be kept in mind to decide which approach is best for a specific target peptide. Both approaches are based on the same principle of attaching the first amino acid to a support and then elongating the sequence by coupling/deprotection cycles with protected amino acids. However, in LPPS, the intermediates are isolated by precipitation and the convergent synthetic strategy is required for obtaining peptides longer than 20 amino acids. Moreover, it must be noted that, although the solvent volume can be reduced >10-fold with respect to standard SPPS, only recently have there been efforts to substitute problematic/toxic organic solvents (e.g., CHCl_3_, DCM, NMP, DFM, THF) with greener alternatives (e.g., EtOAc, CPME), thanks to a new generation of anchors [150]. Hence, continuous investigations further improve the efficiency and greenness of LPPS. The major contributors in this field are companies (namely, Jitsubo, Ajinomoto Bio-Pharma Services, GAPPeptide, Eli Lilly, and Exactmer) that protected their inventions with patents and trademarks, such as molecular Hiving™, Ajiphase^®^, GAP, and PEPSTAR^®^. An overview of the similarities and differences between the two methods is reported in Table 1.

In 2013, Okada et al. exploited the combination of SPPS and LPPS for the synthesis of bivalirudin, a potent and selective inhibitor of thrombin approved by the FDA as an anticoagulant [151]. Following a convergent synthetic approach, fragments of six or seven amino acid residues were prepared with conventional SPPS and then coupled together into the final 20-mer structure with LPPS using a hydrophobic *ortho-*, *para*-substituted benzyl alcohol anchor, such as those commercialized by Molecular Hiving^TM^ (Figure 23). In 2019, the same group used similar anchors for a full LPPS, 100 g scale synthesis of the 10-mer peptidomimetic icatibant, a bradykinin receptor antagonist used for the treatment of hereditary angioedema (HAE) [152]. More recently, Yeo et al. reported the synthesis of enkephalin-like peptides and two analogs of the API octreotid (i.e., octreotate amide and octreotate) by means of LPPS [153]. In this case, the authors employed a pepstar anchor (Figure 23) and organic solvent nanofiltration (OSN) for isolation of the intermediates and the final product to achieve a very efficient one-pot liquid phase synthesis.

### 3.4. Chemoenzymatic Peptide Synthesis (CEPS)

Over the past decades, biocatalysis has gained increasing consensus in the scientific community and is now considered one of the milestones of green chemistry. The advantages of biocatalysis include excellent regio- and chemoselectivity, mild reaction conditions, and low waste generation, which make this approach highly attractive for several academic and industrial applications, including drug discovery, development, and manufacturing [154,155,156]. Chemoenzymatic peptide synthesis (CEPS) exploits different classes of enzymes for the synthesis of therapeutical peptides. In this context, the most popular biocatalysts comprehend sortases, butelases, and subtiligase variants (namely, peptiligase and omniligase). Their applicability is currently broadening thanks to advances in recombinant technology and the development of cutting-edge techniques to adapt an enzyme for the desired application. Such engineered enzymes are tailored for a specific substrate, enabling high activity and specificity while improving the biocatalyst’s stability [157,158].

**Figure 24:**
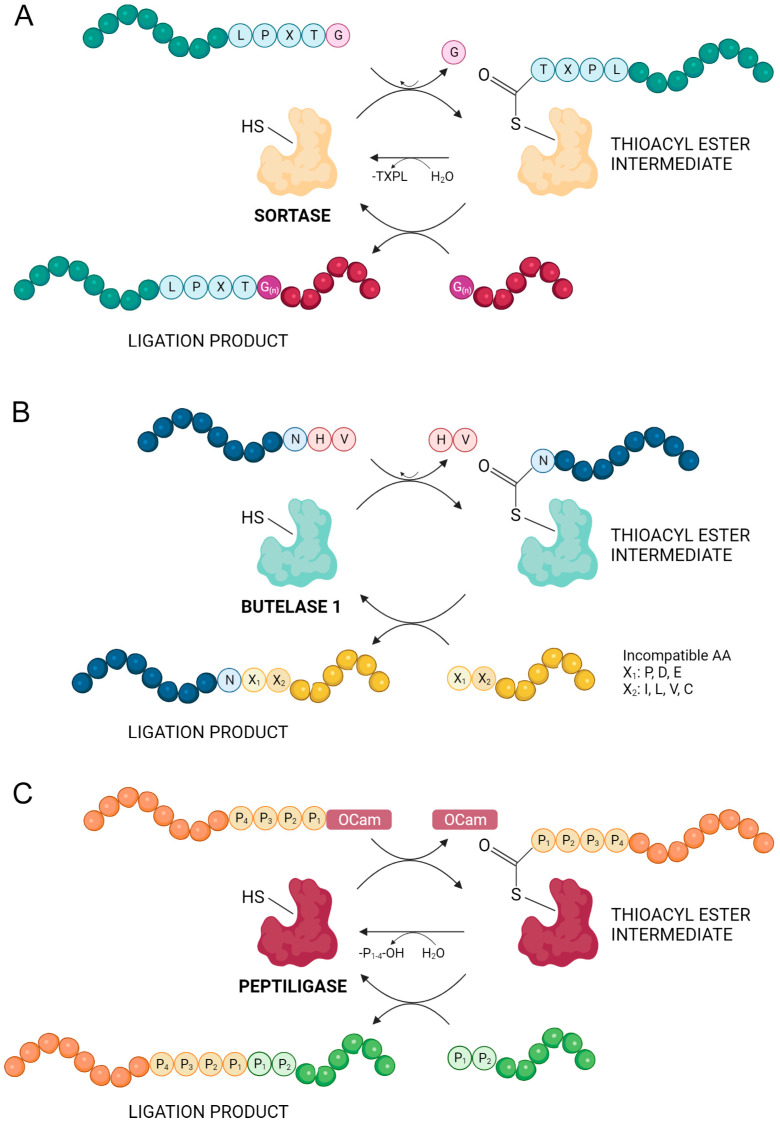
A schematic representation of peptide ligation by cysteine-dependent enzymes, such as sortase (**A**), butelase1 (**B**), or peptiligase (**C**). Picture adapted from reference [105].

Sortase A (SrtA) is one of the most popular, commercially available, and robust enzymes (Figure 24). It has been used for a variety of applications, from peptide synthesis and protein conjugation to C-terminal labeling, protein immobilization, and peptide cyclization. SrtA exploits an LPXTG recognition motif (where X can be any amino acid) to cleave the C-terminal glycine and form a thioacyl-enzyme intermediate. This is coupled with the N-terminal amino group of the acyl acceptor peptide, which must be a glycine residue. Such requirements in both the acyl donor and acceptor fragments make this process—called sortagging—highly selective, but they also limit the substrate scope, since the LPXTG motif sequence must be part of the target peptide (Figure 24). Furthermore, SrtA has a low catalytic efficiency, meaning that a higher amount of enzyme is needed to achieve good conversions. To overcome such limitations, engineered Sortase variants have been studied [157]. As a more recent and promising alternative to Sortases, asparaginyl endoproteases (AEP) are quickly becoming a pillar of CEPS. Within this class of enzymes, Butelase 1 (Figure 24A) deserves to be mentioned. Similarly to SrtA, the first step consists of the formation of a thioester acyl-enzyme intermediate, but in this case, the recognition sequence on the acyl donor is a shorter NHV motif, of which only an Asp residue is conserved in the ligated peptide. Conversely, any amino acid is tolerated in the N-terminal first position (except Pro, Asp, and Glu), but in the second position, only Ile, Leu, Val, or Cys are compatible. Butelase 1 is also endowed with much higher catalytic efficiency than sortases (0.005 molar equiv. are sufficient) and typically gives higher yields (Figure 24B). This enzyme is naturally more efficient in cyclization reactions, but it has also been used with success in intermolecular ligation. Its versatility and efficiency, as well as the development of new variants, make Butelase 1 suitable for a wide range of applications (e.g., head-to-tail macrocyclization, the modification of live cell bacterial surfaces, the synthesis of peptide dendrimers and protein conjugates) [157].

Finally, Subtilisin protease is the progenitor of another class of extremely valuable biocatalysts. Several variants were obtained by genetic engineering since its discovery, eventually leading to a highly efficient and versatile enzyme named omniligase-1 [159]. Its substrate scope is broad, with only two limitations on the acyl donor peptide: the P_4_ position requires hydrophobic or weakly polar amino acids, while in the P_1_ position, proline is not accepted. Conversely, proline is not tolerated both in the P_1′_ and P_2′_ positions of the acyl acceptor. The only other requirement is that the C-terminal fragment must bear an activated ester, typically the carboxyamidomethyl (OCam) ester (Figure 24C). The potential of omniligase-1 in the production of peptide drugs was recently demonstrated by Pawlas and coworkers, with the multi-gram scale synthesis of exenatide, a 39-mer therapeutic peptide used in the treatment of diabetes type II. In this approach, the acyl acceptor and the activated acyl donor were obtained through standard SPPS and ligated, as unprotected fragments, in the last step with high catalytic activity. The overall yield was almost doubled in respect to conventional fully SPPS methods, and the product was obtained within pharmacopeia specifications [160]. This demonstrates that optimal results can be achieved when integrating CEPS with other technologies (such as SPPS and LPPS) in a fragment condensation approach. In fact, enzymes are suitable for coupling unprotected peptide fragments in aqueous media with high efficiency, overcoming solubility issues related to conventional chemical fragment condensations.

## 4. Conclusions

Peptides have emerged as a revolutionary class of therapeutic agents, redefining the landscape of contemporary pharmaceuticals. Their unique biochemical properties, coupled with their target specificity and potency, have positioned them uniquely, bridging the gap between small molecules and biologic drugs. Over 80 therapeutic peptides have been approved to treat a wide array of diseases, ranging from infectious diseases, cardiovascular, dysmetabolic diseases, and cancer. Furthermore, hundreds of peptides are undergoing preclinical studies and clinical development. The expanding interest from both academia and pharmaceutical sectors in peptide-based therapies is evident from the exponential rise in scientific publications and patents over recent years. Given their therapeutic potentials, market prospects, and economic values, it is expected that therapeutic peptides will continue to attract investment and research efforts. As we celebrate these milestones, the synthesis of peptides still remains a significant challenge, especially when viewed through the lens of green chemistry. The global push towards sustainable practices has witnessed both academic and industrial sectors advancing towards more eco-friendly peptide synthesis and purification techniques. The call to action is clear: academic research groups must accelerate in finding new greener up-scaling in their green peptide synthesis methodologies, and the pharmaceutical industry must be proactive in investing in these greener approaches. Emerging technologies, such as water-based SPPS, LPPS, microwave-assisted peptide synthesis, and CEPS, together with the discovery and exploitation of green solvent and coupling agents, hint at a green transition in this field and will likely gain prominence, addressing the growing manufacturing demand. While the path to the ideal green peptide synthesis has probably still to be traced, the current knowledge and technological advancement are bringing us significantly closer to the goal.

## Figures and Tables

**Table 1:** Similarities and differences between LPPS and SPPS. Parameters taken into account for comparison of the two techniques are report in bold in the first column on the left.

	Liquid-Phase Peptide Synthesis (LPPS)	Solid-Phase Peptide Synthesis (SPPS)
**Medium**	Solution	Insoluble polymer
**Batch size**	Any (usually large scale)	Any (usually small scale)
**Timing**	Slow	Fast
**Automation**	Difficult	Semi- or fully-automated
**Synthetic strategy**	Generally convergent	Stepwise
**Protecting groups employed**	Typically Boc or Z	Typically Fmoc
**Side-chain protection**	Minimum	Maximum
**Consumption of AA**	Moderate	High
**In-process analysis**	Direct monitoring (e.g., HPLC)	Indirect monitoring
**Purification of intermediates**	Possible (usually by precipitation)	Not possible
**Final purification**	Relatively simple	Laborious

## Data Availability

No new data were created or analyzed in this study. Data sharing is not applicable to this article.
